# Development of Teleoperated Robotic System for Remote Intraocular Microsurgery

**DOI:** 10.1002/advs.202509849

**Published:** 2025-11-10

**Authors:** Andi Xu, Zhangkai Lian, Wenben Chen, Yanlin Li, Jiali Liu, Hongli Liang, Rihui Song, Zikai Lin, Mingyuan Li, Yining Huang, Ling Jin, Zheming Zhang, Xiaoyue Wei, Bingqian Liu, Wei Ma, Tao Li, Hang Shao, Wei Liu, Kun Gao, Xin Zhang, Pisong Yan, Ruiyang Li, Ruixin Wang, Xiaohang Wu, Duoru Lin, Xiang Chen, Jinghui Wang, Xingwu Zhong, Mohammad Ali Nasseri, Kai Huang, Haotian Lin

**Affiliations:** ^1^ Zhongshan Ophthalmic Center Sun Yat‐sen University WHO Collaborating Centre for Eye Care and Vision State Key Laboratory of Ophthalmology Guangdong Provincial Key Laboratory of Ophthalmology and Visual Science Guangdong Provincial Clinical Research Center for Ocular Diseases Guangzhou 510623 China; ^2^ School of Computer Science and Engineering Sun Yat‐sen University Guangzhou 510006 China; ^3^ School of Artificial Intelligence Sun Yat‐sen University Zhuhai 519082 China; ^4^ Jiaxing Key Laboratory of Visual Big Data and Artificial Intelligence Yangtze Delta Region Institute of Tsinghua University Jiaxing 314006 China; ^5^ Hainan Eye Hospital and Key Laboratory of Ophthalmology Zhongshan Ophthalmic Center Sun Yat‐sen University Haikou 570311 China; ^6^ School of Medicine and Health Technical University of Munich 81675 Munich Germany; ^7^ Department of Genetics and Biomedical Informatics Zhongshan School of Medicine Institute for Frontier Interdisciplinary Research in Health Sciences and Technology Sun Yat‐sen University Guangzhou 510080 China

**Keywords:** intraocular surgery, remote microsurgery, surgical robot, telemedicine

## Abstract

Microsurgery has revolutionized modern surgery through its precision and favorable outcomes, but it remains limited by operational difficulty and the scarcity of skilled surgeons. Advances in robotics and communication technology have enabled the development of robotic telesurgery, with the goal of eliminating healthcare disparities. However, insufficiencies regarding system precision, surgical image transmission quality, and operational latency have made remote microsurgery challenging, particularly in intraocular microsurgery. A teleoperated robotic system is presented, featuring micrometer‐scale precision and remote center of motion design, to ensure safety and flexibility within the confined operating space of the vitreous cavity. To validate its feasibility and safety, a randomized multicenter study is conducted involving in vivo subretinal injections in 51 pigmented rabbits. Surgeons using teleoperated system achieve nearly twice the first‐attempt success rate and significantly fewer surgical complications than manual surgery. Furthermore, the system's versatility is showcased by successfully removing microscale intraocular foreign bodies in 15 porcine eyes and the communication stability is validated in multicenter remote surgeries, including procedures across the Qiongzhou Strait. These findings establish the system's safety, reliability, and versatility for performing remote intraocular procedures, highlighting its potential of remote microsurgery to improve surgical outcomes and broaden access to specialized care.

## Graphical abstract

A teleoperated robotic system, characterized by micrometer‐scale precision and a remote center of motion design, is developed and validated to ensure the safety and flexibility of remote intraocular surgery. This system exhibits superior performance in experimental evaluations and holds significant potential for advancing remote microsurgery—specifically, to enhance surgical outcomes and expand access to specialized ophthalmic care for underserved populations.

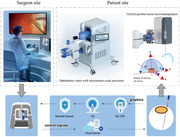

## 1 Introduction

In recent decades, the field of surgery has undergone a substantial revolution with the advent of microsurgery, which has demonstrated advantages such as high precision, minimal invasiveness, and favorable postoperative outcomes.^[^ ^1^ ^]^ Rapid advancements in microscopy and the miniaturization of surgical instruments have enabled surgeons to visualize intricate anatomical structures and perform precise operations while better protecting adjacent tissues, thereby reducing surgical trauma.^[^ ^2^, ^3^ ^]^ These microsurgical techniques not only lower surgical risks and improve postoperative outcomes but also make previously challenging procedures, such as delicate brain and retinal surgeries, more feasible. However, microsurgery demands a high level of proficiency from surgeons and requires a longer learning curve.^[^ ^4^ ^]^ Globally, qualified surgeons for microsurgical procedures are scarce and unevenly distributed.^[^ ^5^ ^]^ Furthermore, even experienced surgeons struggle to overcome physiological tremors to achieve steady and precise microsurgical operations.^[^ ^6^, ^7^ ^]^ These obstacles significantly hinder the adoption of microsurgery, particularly in low‐ and middle‐income countries (LMICs).^[^ ^8^, ^9^ ^]^

Intraocular surgery is a critical aspect of ophthalmic microsurgery, requiring exceptional precision and steadiness.^[^ ^10^, ^11^ ^]^ Subretinal injections, for example, are highly intricate procedures that involve injections of medications through a microneedle delicately inserted between the neural layer and the retinal pigment epithelium (RPE) layer of the retina at a depth of 150‐200 µm, pushing the boundaries of human dexterity.^[^ ^12^ ^]^ This technique is an important modality for treating submacular hemorrhage and for gene therapy, but even slight errors can lead to severe complications, underscoring the challenges of this surgery.^[^ ^13^ ^]^ While the ever‐increasing prevalence of retinal and vitreous disorders—affecting millions annually—has led to an increased demand for vitreoretinal surgery, the limited number of qualified surgeons and their extensive training periods are significant obstacles that must be overcome.^[^ ^5^, ^14^ ^]^

In recent years, rapid advancements in telecommunication technologies and surgical robotics have paved the way for the possibility of remote robotic surgery.^[^ ^15^, ^16^, ^17^ ^]^ Currently, with the integration of next‐generation communication technologies, robotic surgical systems such as the da Vinci system have enabled surgeons to perform remote laparoscopic procedures with remarkable stability and precision.^[^ ^18^, ^19^ ^]^ The implementation of these telesurgery systems holds significant potential for overcoming barriers related to surgical precision and stability, transcending the spatial limitations of medical resources. However, conducting remote intraocular surgery, particularly vitreoretinal surgery, presents numerous challenges. The extremely delicate structure of the retina requires exceptionally high precision and stability from robotic systems. Therefore, success in these surgeries relies on clear visualization of the fine details of the retina and an acute sense of depth perception. These requirements impose high standards on the acquisition and transmission of 3D surgical images during telesurgery; even slight system latency or distortions in surgical displays can severely impact the procedures, potentially compromising the effectiveness and safety of the surgery. Furthermore, surgeons typically perform vitreoretinal surgery through extremely small puncture sites on the sclera, meticulously maneuvering surgical instruments within the confined vitreous cavity—a challenge for most robotic surgical systems to replicate to date.^[^ ^20^ ^]^

In this study, we designed a remote robotic system that incorporates a hybrid parallel‐serial micromanipulator^[^ ^21^ ^]^ (HPSM) equipped with a remote center of motion (RCM) design to enable intricate ophthalmic operations in confined intraocular spaces. The HPSM integrated a software‐based adjustable RCM design, distinguishing it from previously reported ophthalmic robots.^[^ ^11^, ^13^, ^22^ ^]^ This system dynamically and rapidly adjusted the RCM point to adapt to intraocular surgery, enhancing flexibility and effective workspace for remote procedures with stringent accuracy requirements. To accommodate remote ophthalmic microsurgeries, we integrated a tremor filtering algorithm and a magnification scaling algorithm into the system's telesurgery mode and ensured real‐time transmission of 3D surgical videos and maneuvering commands via a fifth‐generation (5G) sliced network. To validate the safety and feasibility of this system for remote vitreoretinal surgery, surgeons at one of three different sites remotely performed subretinal injections on live pigmented rabbit eyes located at one patient site. The success rate, operation time, and incidence of intraoperative and postoperative complications in the remote surgery group were compared with those in the manual surgery group. Additionally, to demonstrate the versatility of the system, surgeons were required to remotely remove foreign bodies from enucleated porcine eyes at different surgical sites. Overall success rate (100.0%) was achieved in all remote procedures, demonstrating that our robotic system can safely and effectively assist surgeons in performing microsurgical operations stably and remotely. These findings represent a breakthrough that transcends geographical barriers in ophthalmic surgery, ushering microsurgery into the remote era.

## 2 Results

### 2.1 Remote Intraocular Surgery System

**Figure 1:**
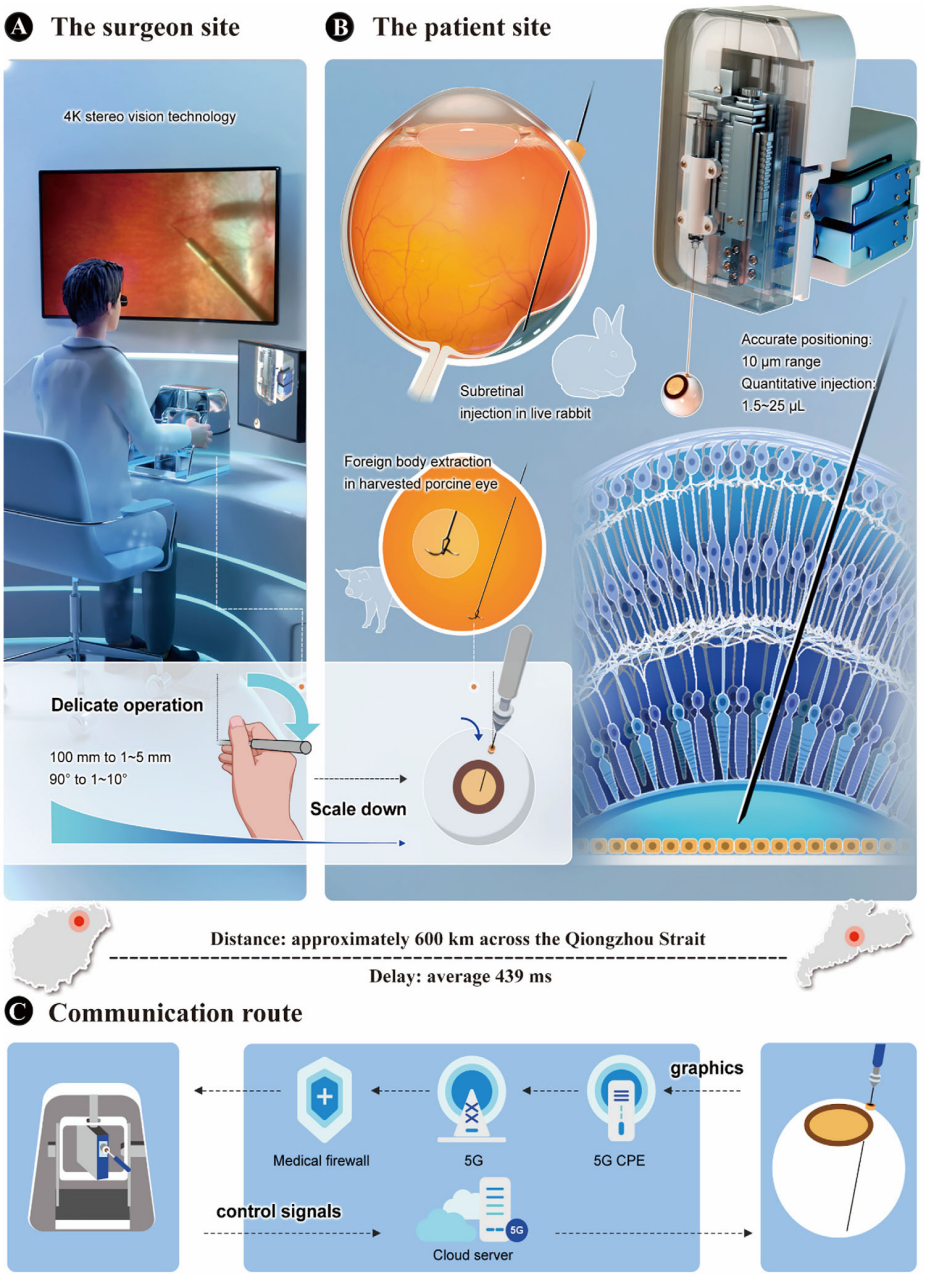
Remote microsurgery system for vitreoretinal surgery. The system is implemented across two sites: A) the surgeon site, where microsurgeons remotely view surgical images and manipulate the robot, and B) the patient site, where the patient and the robot are located. The robot is subjected to software‐based remote center of motion (RCM) with hand tremor filtering and magnification scaling algorithms, achieving an accuracy of 10 µm for single‐depth movement. C) Communication route. A network communication method with parallel transmission of surgical video and robot control commands was used, resulting in an overall average latency of 439 ms between Guangzhou and Haikou.

We developed a remote robotic system to assist surgeons in performing remote intraocular surgery. This system was distributed across two sites: the surgeon site (**Figure** **1**A), which contained the remote controller, surgical robot pedal, and digital stereoscopic display and the patient site (Figure 1B), which contained the subject, surgical microscope, and robotic system. The microsurgeons operated the controller and pedal at the surgeon site to remotely manipulate the robot at the patient site. Surgical videos were captured via stereoscopic cameras integrated with the surgical microscope (Zeiss OPMI VISU 200) and presented to the microsurgeons via a digital stereoscopic display, which provided ultrahigh‐definition videos with great depth of field, resulting in the clear visualization of retinal tissue. Remote transmission of the surgical view was facilitated by a 5G sliced network linked to the hospital's firewall, while the remote robotic control commands were transmitted over a 5G network integrated with cloud servers (Figure 1C).

### 2.2 Framework of the Robotic System

**Figure 2:**
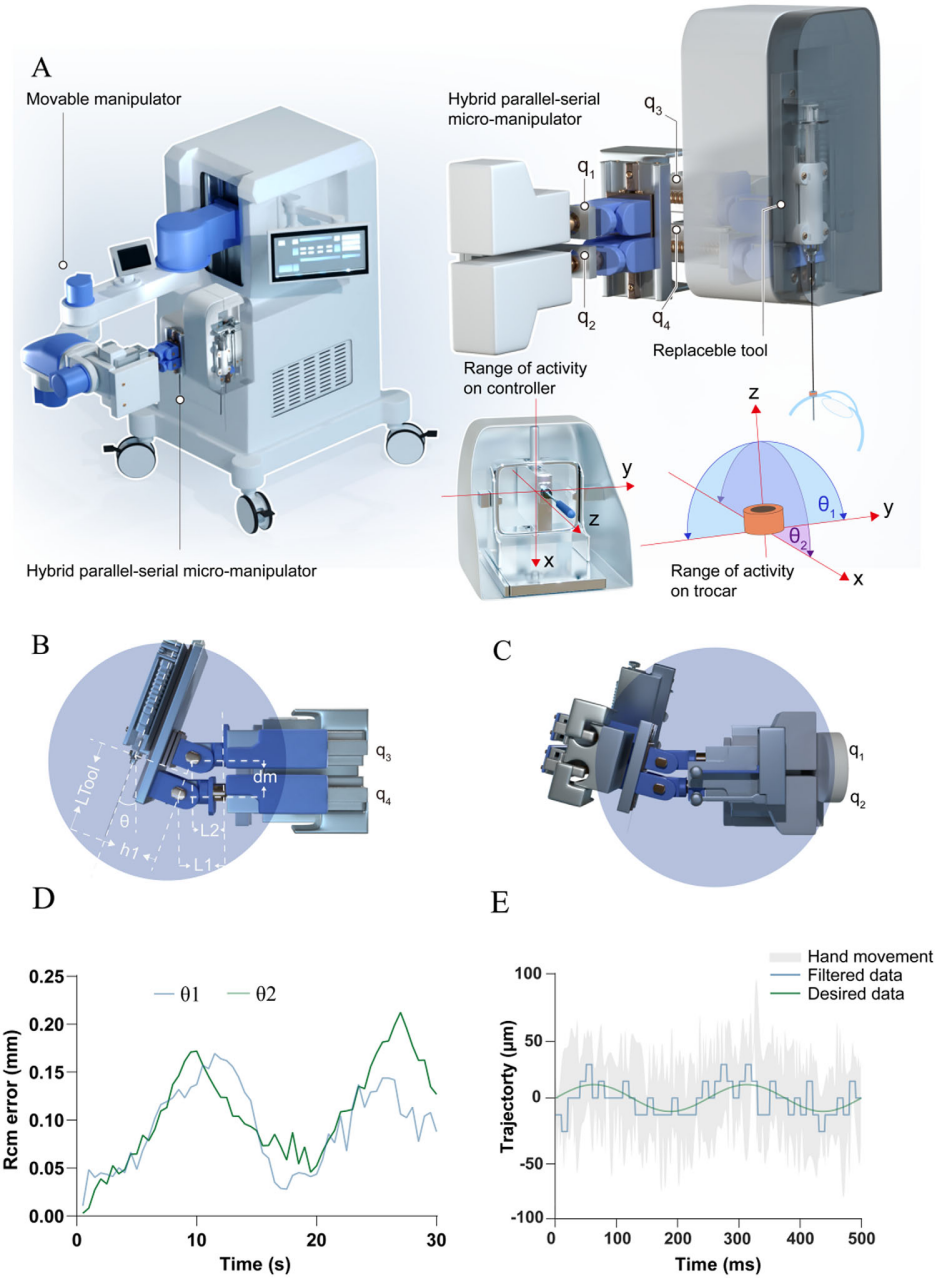
Characterization of the remote robotic system. A) Hybrid parallel‐serial micromanipulator with degrees of freedom. B,C) RCM point and movement locus. D) The green and blue lines show the mean RCM errors at two angles (*θ*1 and *θ*2), respectively. E) Performance of the tremor algorithm for 20 µm motion manipulation. RCM, remote center of motion.

Ophthalmic microsurgery is characterized by a confined operating space and a high demand for precision, especially in vitreoretinal surgery where precision down to the micrometer level is crucial. To meet these requirements, we designed a surgical robot controlled through a master‒slave interface featuring a HPSM that combined elements of both parallel and serial manipulator structures to provide greater flexibility and precision in controlling the position and orientation of objects or tools at a very small scale. Unlike previous hardware‐based RCM designs used in other ophthalmic robots, our approach utilized a software‐based RCM design to facilitate precise instrument movements around the scleral incision. This approach not only prevents potential harm to the eyes from lateral instrument translation but also improves surgical flexibility and overall performance within the limited vitreous cavity (**Figure** **2**A; see Video S1, Supporting Information). To accommodate the surgical environment, our robotic system was equipped with a base that secured and mobilized the entire setup while integrating all components. An auxiliary arm provided stable positioning and angular adjustment of the robot and surgical instruments. A control interface displayed motor status and allowed switching between the auxiliary arm and the robotic arm. The robotic arm boasted five degrees of freedom (DOFs), including three translation degrees (X, Y, and Z) and two rotation degrees (θ_1_ and θ_2_) (Figure 2B,C). When operating under RCM conditions, we restricted the translation DOFs along the tangent plane to minimize the risk of injury. In this model, the mean (standard deviation [SD]) RCM errors were 0.09 (0.04) mm and 0.11 (0.05) mm for θ_1_ and θ_2_, respectively (Figure 2D), meeting the requirements for surgical safety.^[^ ^23^ ^]^ Furthermore, we conducted a representative trajectory tracking experiment on a 0.25 mm×0.25 mm square to compare the accuracy and stability between manual and robot‐based movements (Video S2, Supporting Information). The results reveal that the movements of the remote robotic system were more precise and stable than hand movements (Figure S1, Supporting Information).

Furthermore, in ophthalmic microsurgery, an insufficient workspace could lead to situations where the workspace fails to fully cover the target area. Therefore, we increased the intraocular workspace by incorporating an additional motor. This motor was integrated at the junction between the surgical arm and the auxiliary arm, which was aligned parallel to the tool axis in its initial state to provide axial tool insertion and retraction capability. A simulation was conducted based on an adult eyeball size, utilizing a Monte Carlo method with 20 000 sample points. The results show that with the assistance of the auxiliary motor, the coverage rate of the macular region increased from 57.33% to 100% (Figure S2, Supporting Information).

To ensure suitable maneuverability, the motion controller was equipped with a tremor‐filtering algorithm to accurately replicate the surgeon's movements while filtering out physiological hand tremors to further increase surgical precision (Figure 2E). Additionally, we implemented an adjustable motion scaling mechanism that can smoothly convert human hand movements into the robotic arm's fine movements, down to as small as 1/30 of the original hand motion, achieving precision that surpasses traditional manual retinal surgery. Furthermore, the system can personalize robot parameters according to the surgeon's preferences, enabling precise, quantitative, and controlled injection speeds that are difficult to achieve with traditional manual operations.

### 2.3 Workflow of the Validation Study

To assess the feasibility and safety of remote vitreoretinal surgery, we conducted a multicenter animal experiment (Figure 1B). Subretinal injections, which require extreme precision, were selected to test our robotic system. A total of 51 pigmented rabbits were randomly assigned to either the remote group (*n* = 27) or the manual group (*n* = 24). Each rabbit received a 50 µL subretinal injection of balanced salt solution (BSS) via a 41G microneedle in only one eye, which was administered either through a remote robotic procedure or a manual procedure. The rabbits in the remote group were further randomly divided into three subgroups, with each subgroup undergoing surgery performed by surgeons at one of three different sites: the Hainan Eye Hospital (HEH) in Haikou, Hainan Province; the Center for Innovative Diagnostics and Therapeutics in Bio‐Island District (BD) in Guangzhou, Guangdong Province; and the Zhongshan Ophthalmic Center (ZOC) Zhujiang New Town Hospital (ZNTH) in Guangzhou, Guangdong Province. All rabbits underwent baseline fundus photography and optical coherence tomography (OCT) scans to confirm the absence of preoperative ocular diseases. Four senior surgeons from ZOC were randomly divided into two groups. Two surgeons in the manual group performed the surgeries manually, whereas the remaining two surgeons in the remote group conducted the robotic operations remotely.

The workflow of robotic surgery is similar to that of manual surgery, with the primary distinction being that the instruments are controlled remotely by microsurgeons in the former. These surgeons manipulate the controller and pedal to remotely operate the robotic arm at the patient site, enabling precise movement and quantitative injection. To maintain consistent rabbit rearing environments, surgical conditions, and postoperative observations, both the manual group and the remote group received subretinal injections at the same location: ZOC Ouzhuang Hospital (ZOCOH) in Guangzhou, Guangdong Province. Surgeon sites were established at the three abovementioned centers to demonstrate the safety and stability of the remote robotic system across different distances and locations. The study was approved by the Institutional Animal Care and Use Committee (IACUC) of ZOC (O2023001).

### 2.4 Comparison of Remote Robot‐Based and Manual Subretinal Injections

**Figure 3:**
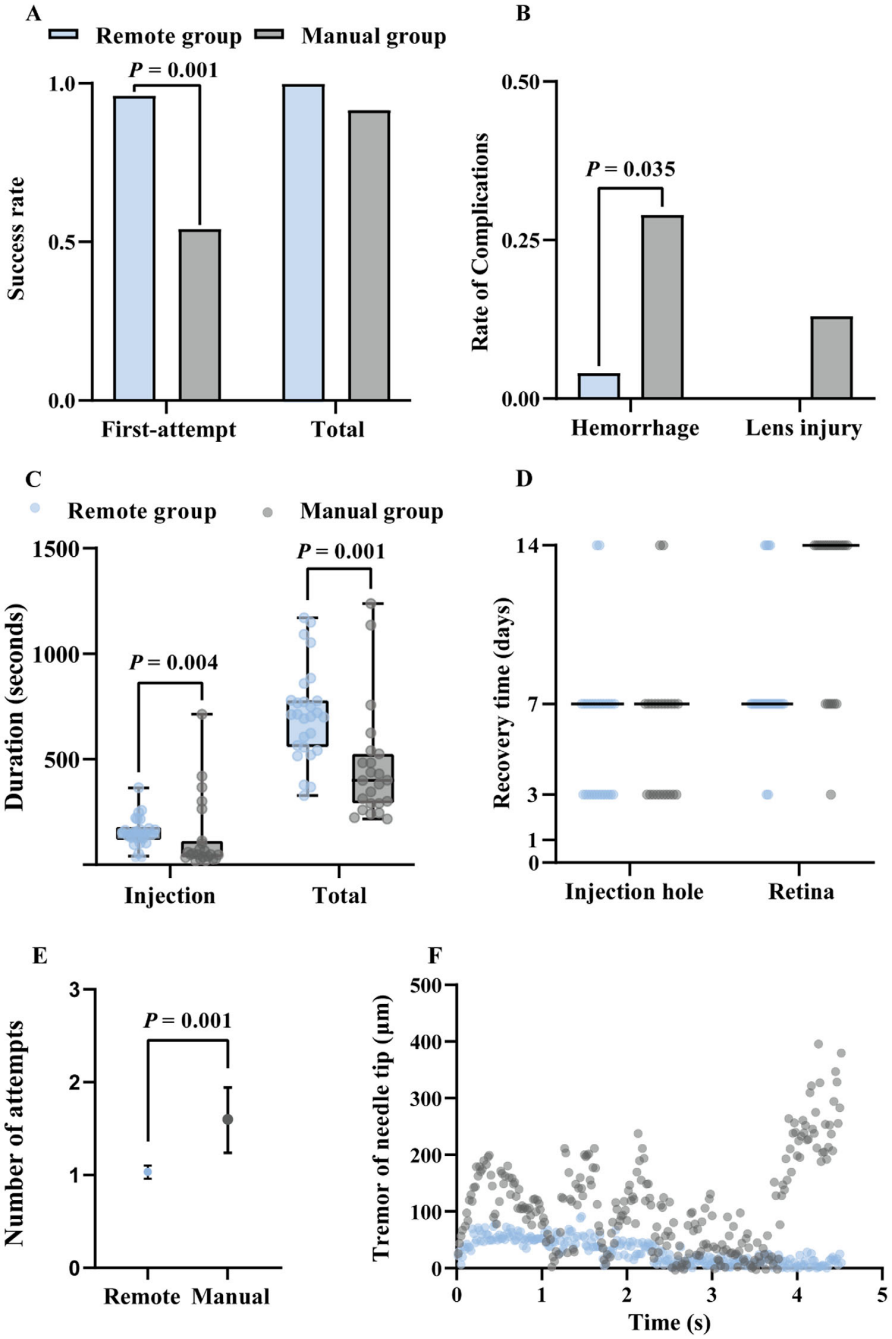
Comparison of remote robot‐assisted vs manual subretinal injection. A) Success rate of subretinal injection in the remote and manual groups. First attempt success is defined as puncturing the retina only once to form an injection hole and completing the subretinal injection during the same procedure. Data presented as rate, *n* = 51, *P*‐values are calculated using Z test. B) Rates of intraoperative complications in the two groups. Data presented as rate, *n* = 51, *P*‐values are calculated using Pearson's chi‐squared test with continuity correction. C) The mean duration of injection and total surgery time. Data presented as mean ± IQR, *n* = 51, *P*‐values are calculated using Mann‒Whitney U test. D) Postoperative retinal recovery observed at different intervals. E) Number of attempts at subretinal injections in the remote and manual groups. Data presented as mean ± 95% CI, *n* = 51, *P*‐values are calculated using Mann‒Whitney U test. F) Tremor of the needle tip during subretinal injections.

The first‐attempt success rate for the subretinal injections in the remote robotic group was significantly greater than that in the manual group (96.3% [26/27] vs 54.2% [13/24]; rate difference, 42.1%, 95% confidence intervals [CI] 21.0% to 63.3%, *P* < 0.0001). The overall success rate was not significantly different between the remote group and the manual group (100% [27/27] vs 91.7% [22/24]; rate difference, 8.3%, 95% CI ‐2.7% to 19.4%, *P* > 0.05) (**Figure** **3**A; see Video S3, Supporting Information).

Two types of intraoperative complications were observed in the video recordings: hemorrhage and lens injury. Hemorrhage was defined as retinal bleeding caused by contact with the retina, whereas lens injury was defined as any contact with or impairment of the lens. The incidence of hemorrhage in the remote group was significantly lower than that in the manual group (3.7% [1/27] vs 29.2%, [7/24]; rate difference, ‐25.5%, 95% CI ‐45.0% to ‐5.9%, *P* = 0.035). Lens injury was observed in only 3 rabbit eyes in the manual group (12.5% [3/24]) (Figure 3B).

The mean duration of surgery, defined as the interval from inserting the first trocar into the sclera to completing the injection and removing the last trocar (measured in minutes and seconds), was 11:54 (interquartile range [IQR] 3:42) for the remote group and 7:39 (IQR 3:56) for the manual group (*P* = 0.001) (Figure 3C). The median time required to move the 41G needle from the trocar and successfully complete the subretinal injection was longer in the remote group than in the manual group (2:33 [IQR 0:47] vs 2:02 [IQR 1:19], *P* = 0.004).

Recovery time and postoperative complications were evaluated via fundus photography and OCT imaging (Figure S3, Supporting Information). On the seventh day after surgery, a significantly higher percentage of rabbits in the remote group achieved retinal recovery compared with the manual group (85.2% [23/27] vs 29.2% [7/24], *P* < 0.0001). No significant differences were observed for the recovery time of injection hole between two groups on the seventh day after surgery (remote robotic group 92.6% [25/27] vs manual group 87.5% [21/24], *P* > 0.05) (Figure 3D), but the remote group had significantly fewer attempts of injections than the manual group did (*P* = 0.001) (Figure 3E).

Additionally, we developed a tremor recognition algorithm with visualization features to objectively assess the tremor of the needle tip during surgery between the remote group and the manual group (Video S4, Supporting Information). The remote group exhibited a significantly lower tremor amplitude (mean ± SD, 29.13 ± 1.32 µm) compared with the manual group (115.11 ± 4.98 µm) (*P* < 0.0001; Figure 3F).

### 2.5 Instrumentation Versatility of the Remote Robotic System

**Figure 4:**
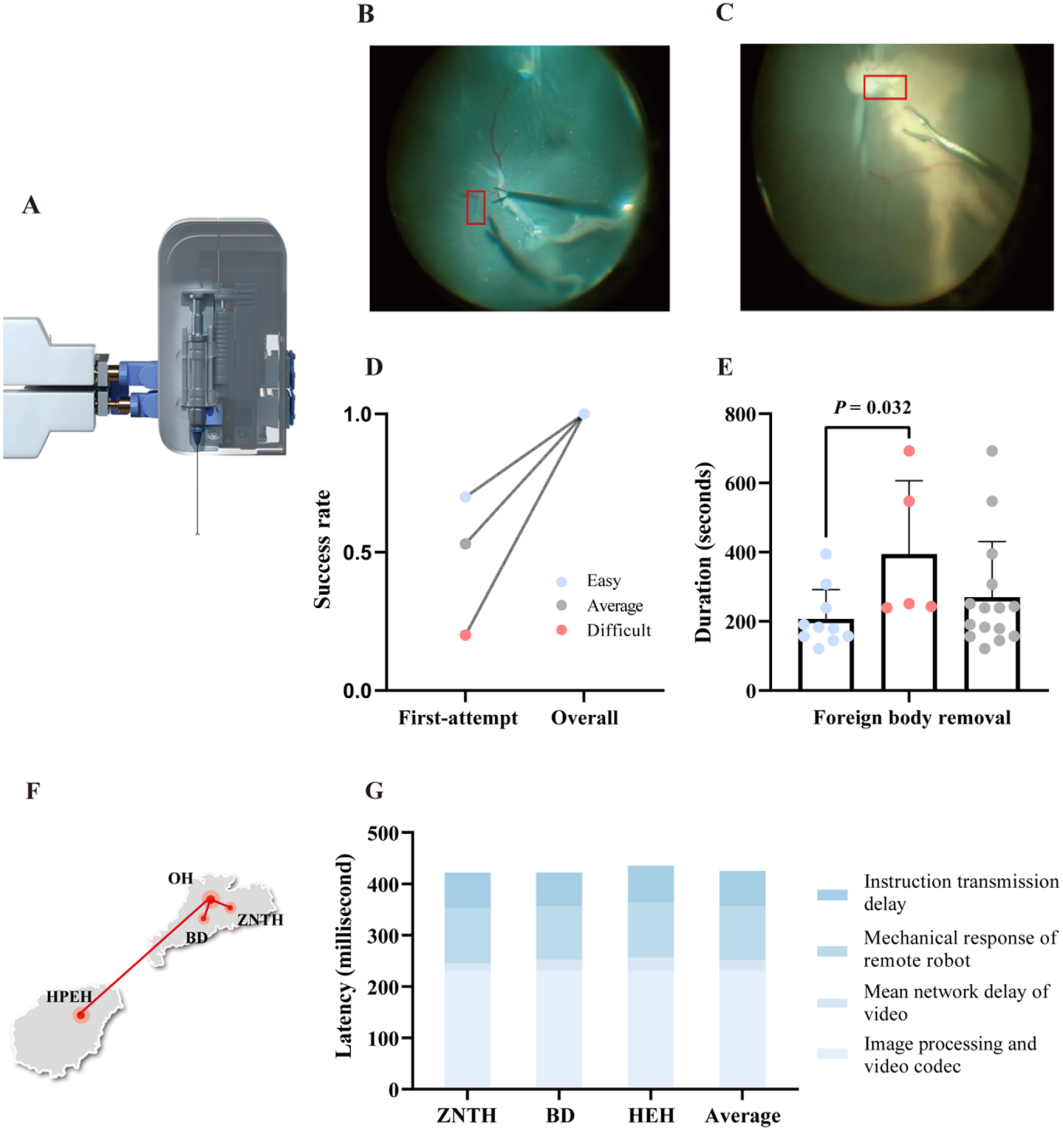
Instrumentation and multicenter feasibility analysis of remote robot‐assisted microsurgery. A) Robotic arm equipped with customized retinal forceps. Feasibility assessment of remote foreign body removal at two levels of difficulty: B) easy (suture on the flat part of the retina) and C) difficult (suture on a retinal fold). D) Success rates for the remote foreign body removal at easy and difficult levels. E) The mean duration of foreign body removal at two levels. Data presented as mean ± SD, n=15, *P*‐values are calculated using Mann‒Whitney U test. F) Multicenter feasibility. To verify the geographical applicability of the remote robotic system, remote control was executed from three locations with varying distances from the patient site of approximately 3 km (ZNTH), 20 km (BD), and 600 km across the Qiongzhou Strait (HEH). G) Latency and components. The average overall latency, including the instruction transmission delay, mechanical response of the remote robot, and network delay of the video, image processing and video codec at the three sites was 429 ms. The latency was relatively consistent across the three locations (ZNTH: 422 ms, BD: 426 ms, HEH: 439 ms). ZNTH, Zhujiang New Town Hospital; BD, Bio Island; HEH, Hainan Eye Hospital.

The robotic arm of the remote system was designed to accommodate a diverse array of microscopic instruments suitable for various clinical scenarios. Thus, to validate the robot's instrumentation versatility, we specifically conducted assessments focused on the feasibility of remote robotic foreign body removal with the proposed system. In this procedure, the surgeons were required to remotely control retinal forceps integrated into the robotic arm (**Figure** **4**A) to remove a 2 mm long suture (Alcon, 10–0 nylon suture) positioned near the optic disc of 15 enucleated porcine eyes (Video S5, Supporting Information). We categorized foreign body removal into two groups: easy (suture on the flat part of the retina, *n* = 10, Figure 4B) and difficult groups (suture on a retinal fold, *n* = 5, Figure 4C). The first‐attempt success rate was 70.0% (7/10) in the easy group and 20.0% (1/5) in the difficult group. Overall, the success rate was 100% in all 15 porcine eyes, with an overall first‐attempt success rate of 53% (Figure 4D). The duration of foreign body removal was significantly longer in the difficult group (mean ± SD, 6:34 ± 3:32 vs 3:33 ± 1:29, *P* = 0.032; Figure 4E). Since foreign body removal was carried out on ex vivo porcine eyes, no complications were recorded during or after the operation.

### 2.6 Feasibility and Stability of Multicenter Robotic Microsurgery

To assess the geographical applicability of the remote robotic system, we conducted surgeries with surgeons located 3 km, 20 km, and 600 km (notably across the Qiongzhou Strait) from the patient site (Figure 4F). The first‐attempt success rate at these locations was consistently high (HEH: 90.9% [10/11], BD: 100% [8/8], ZNTH: 100% [8/8]). Remote subretinal injection was completely successful without significant complications. All remote foreign body removals were successfully accomplished. Furthermore, no instances of video or surgical interruptions occurred throughout the procedures, regardless of the geographic distance between the surgeon and patient sites. The system latency represented the sum of the instruction transmission delay, mechanical response delay of the remote robot, and network delays associated with video transmission, image processing, and video codec. The average overall delay (the time required for motion commands to be transmitted from the remote console to the robot and back to the digital screen) across the three centers was 429 ms, with relatively consistent performance among the three locations (ZNTH: 422 ms, BD: 426 ms, HEH: 439 ms). This delay was deemed acceptable for current surgical requirements on the basis of our results and those of previous studies^[^ ^24^ ^]^ (Figure 4G).

## 3 Discussion

High‐quality vitreoretinal surgical resources are particularly scarce in LMICs, primarily due to a shortage of skilled surgeons. For example, in China, only approximately 1000 surgeons are qualified retinal specialists, all of whom are located in major cities, whereas tens of millions of potential patients are distributed in small‐ and medium‐sized cities.^[^ ^25^ ^]^ Challenges in obtaining transportation to distant surgical centers and time delays increase the difficulty of accessing medical treatment, significantly increasing the cost of care and the risk of blindness.^[^ ^26^, ^27^ ^]^ To address these issues, we designed a remote robotic system tailored for ophthalmic microsurgery, demonstrating for the first time the viability and safety of remote micrometer‐level vitreoretinal surgery in scenarios such as overseas operations. We demonstrated the clinical feasibility of our remote robotic system through controlled, fixed‐volume subretinal injections into rabbit eyes in vivo, in which robotic‐based operations significantly outperformed manual operations in terms of the first‐attempt success rate and avoidance of surgical complications. Additionally, our results demonstrate the system's capability for micron‐level clamping operations through ex vivo porcine experiments, further underscoring the promising potential of our system for a wide range of high‐precision remote operations. The ability of the remote robotic system to be rapidly and flexibly deployed in multiple centers is expected to provide a new pathway for overcoming the geographical disparity in accessing microsurgery resources, thereby promoting greater equity and accessibility. Our results also show that this remote robotic system has great potential in emergency scenarios, such as those characterized by sudden interventional demands, including central retinal artery occlusion and macular hemorrhage, to ensure prompt delivery of emergency ophthalmic services.

Microsurgery, particularly in vitreoretinal applications, poses a formidable challenge due to its requirement for extreme precision at the micrometer level, demanding a training period that often exceeds ten years.^[^ ^28^, ^29^ ^]^ This has led to a global scarcity and uneven distribution of skilled microsurgeons. In response, we have developed a high‐precision surgical robotic system characterized by a unique HPSM, a software‐based RCM design, and flexible remote deployment via master‐slave control to enable remote vitreoretinal operations. The system's tremor‐filtering algorithm adjusts to the physiological tremor amplitudes of the individual surgeon to improve robotic arm accuracy. With an adjustable scaling ratio from 1:1 to 1:30, dynamic motion scaling facilitates the precise control necessary for delicate procedures such as subretinal injection, which is pivotal in the treatment of macular hemorrhage and administration of gene therapy. Moreover, the modular design of the robotic arm enables rapid instrument changes, and allows a diverse set of vitreoretinal operations to be performed. Overall, our findings suggest that telesurgery using this innovative system can outperform traditional manual methods for subretinal injections, effectively reducing the risks of complications and lowering operating costs, particularly in resource‐limited settings. Although the total procedure time was longer in the remote group, the intraocular surgical duration—a key factor influencing clinical outcomes—was comparable between the two groups. The extended total time is likely attributable to the initial positioning of the robotic system, a preparatory step that does not directly affect the critical intraoperative phase. Therefore, the additional time in remote operations may not exert a negative impact on clinical outcomes. The system introduces a novel application for surgical robots in regions with a shortage of skilled microsurgeons, potentially transforming vitreoretinal surgery by overcoming geographical and resource‐related challenges. It offers significant technical support and translational benefits, advancing the World Health Organization (WHO) vision of equitable and accessible eye health for all, especially in LMICs.^[^ ^30^ ^]^

Communication and latency are crucial in remote surgery, as any interruption in the transmission of video or command signals during surgery can increase the risk of irreversible harm. In this study, the 5G network slicing was employed to achieve end‐to‐end transmission, ensuring network isolation to safeguard privacy while maintaining network stability.^[^ ^31^ ^]^ Throughout our study, we did not observe any interruptions in video or surgical operations, even under overseas conditions. The overall latency of our system ranged from 400 to 500 ms, meeting the requirements established in previous studies, where a surgical latency of less than 600 ms was considered acceptable.^[^ ^24^ ^]^ Furthermore, the system's exceptionally high success rate in performing remote operations further demonstrated its stability and acceptability of its latency. In the future, advances in communication technology and arithmetic power, such as 5G deterministic networks with dual transmit/receive module and uplink prescheduling strategies, can further reduce the overall latency of the remote robotic ophthalmic surgery systems.^[^ ^32^ ^]^

Our study had several limitations. First, although human patients were not involved, operating on rabbit eyes presented greater challenges compared to human eyes. Rabbit lenses are larger, and their retinas are thinner, which significantly restricts maneuverability within the intraocular space and makes surgery more difficult. We also employed ex vivo porcine eyes for remote foreign body removal, demonstrating the generalizability of our system. These intentional model selections provide a robust preclinical foundation and represent a critical step toward future clinical application in human patients. Second, we were unable to encompass the full spectrum of fundus surgeries; therefore, we focused on specific retinal procedures that demand high operational precision, including subretinal injections and foreign body removal from the retinal surface. Future developments will allow us to expand the applicability of our instruments to accommodate a broader range of surgical procedures. Third, despite exploring geographical versatility by hosting the robotic system in three different locations, this telesurgery system is currently restricted to using the same carrier within China. Additional experiments are required to validate its ability to perform surgical operations over longer distances, such as across nations or continents. In this study, cross‐strait remote operation has been achieved by leveraging 5G slicing‐based roaming technology, demonstrating technical potential for conducting intercontinental surgery. Further research and exploration are required to truly realize the clinical application of intercontinental surgery.

In conclusion, we developed a high‐precision remote microsurgery system and successfully conducted remote micrometer‐level vitreoretinal surgeries. Our system offers substantial advantages in remote, high‐precision ophthalmic surgery, lowering the surgical threshold and enabling rapid deployment across multiple locations. This system has the potential to overcome geographical constraints in intraocular microsurgery, reduce transportation costs, and improve clinical outcomes, heralding the era of high‐precision telesurgery.

## 4 Experimental Section

### Samples and Ethical Approval

We sought to verify the feasibility and safety of remote vitreoretinal microsurgery via a robotic system tailored to the present study. Pigmented rabbits were selected as the animal model for subretinal injections because their eyeballs are similar in size to those of the human eye. Compared with the human retina, pigmented rabbit eyes have no macular structure but do exhibit a retinal nerve layer thickness of approximately 0.25 mm, which is thinner than that of humans. A total of 51 rabbits (25 males and 26 females), aged between 3 and 5 months and weighing 2.5 to 3.0 kg, were randomly divided into two groups: a manual group (control group) consisting of 24 rabbits and a remote group (experimental group) comprising 27 rabbits. For each rabbit, a single eye was randomly selected to receive subretinal injections. At the end of the observation period, all the pigmented rabbits were humanely euthanized in accordance with the AVMA Guidelines for the Euthanasia of Animals. An overdose of sodium pentobarbital (100 mg kg^−1^) was administered intravenously via the marginal ear vein. Euthanasia was confirmed by absence of a corneal reflex, failure to detect respiration, and absence of a heart beat for a period of more than 5 min. In addition, to assess the instrumentation versatility of the system, 15 enucleated porcine eyes were subjected to foreign body removal from the vitreous cavity via the remote robotic system.

This study was conducted in accordance with the Regulations of the People's Republic of China on the Management of Laboratory Animals and was approved by the IACUC of ZOC.

### Surgeons and Preoperative Training

Four senior surgeons from ZOC with extensive experience in the field of vitreoretinal surgery were enrolled in this study and tasked with performing the procedures either manually or with the remote system. To ensure that the surgeons were proficient with the surgical equipment, procedures, and robotic operations, they underwent initial training in subretinal injections and intraocular foreign body removal. Each surgeon subsequently performed subretinal injections in 2 in vivo rabbits to become familiar with the procedure. Specifically, the surgeons in the remote group were instructed to utilize the robotic system for their training exercises. The samples for training were not included in the formal experiments.

### Multicenter Surgical Settings

For the subretinal injections, remote surgery was conducted through a dual‐setup comprising a local operating room at the patient site and a remote manipulation platform at the surgeon site. The patient site was located in the animal experiment center of the ZOCOH in Guangzhou, Guangdong Province, where the operative object, microscope, and surgical robot were located. Only one patient site was employed, primarily to ensure uniformity in the surgical conditions, the rearing environment of the rabbits, and the postoperative observations. Surgeons at the surgeon site in each of the three centers remotely manipulated the robot to perform the operations. Notably, these sites were located approximately 600, 20, and 3 km from the patient site. For the manual group, all surgeries were conducted in the animal experiment center of the ZOCOH to maintain consistent surgical conditions with those of the remote group except for the utilization of the robot.

### Subretinal Injection Surgical Procedures

After dilating the pupils with eye drops containing 0.5% tropicamide and 0.5% phenylephrine hydrochloride, the pigmented rabbits were sedated with an intramuscular injection of tamsulosin II at a dose of 0.1 mL kg^−1^. They were then anesthetized with an intravenous injection of 2.5% pentobarbital solution at a dose of 25 mg kg^−1^ in the ear margin. The skin and conjunctival sac were disinfected with 5% povidone‐iodine. Two 23 G scleral puncture openings were manually created by a surgical assistant and positioned 2 mm from the corneal limbus; one puncture was made for the ceiling fiber optic illumination, and the other was made for insertion of a 41 G microinjection needle. Subsequently, subretinal injections were conducted under a Zeiss OPMI VISU 200 operating microscope equipped with a 120° wide‐angle lens. In the manual group, the surgeons used a microinjector to perform retinal punctures manually and introduced 50 µL of BSS into the subretinal cavity with an Alcon Constellation in VFC mode. In the remote group, the syringe containing the BSS was attached to the surgical robotic arm. A surgical assistant at the patient site (ZOCOH) activated the robot's localization mode (docking mode) to conduct preoperative preparations. First, the auxiliary robotic arm was wirelessly maneuvered close to the scleral puncture port. The front end of the injection needle was then inserted into the trocar, which served as the RCM point for the surgical robot's control system. At this stage, the surgical robot transitioned to retinal telesurgery mode under manipulation from the lead surgeon, which was located at the surgeon site. Under the surgical field of view transmitted in real time through the remote digital stereoscopic display system, the lead surgeon remotely managed the movement of the injection needle into the vitreous cavity with the controller. After the needle reached the retina, it was gradually introduced into the subretinal area by moderating the needle speed through motion scaling. The surgical instrument could then be held steady in a stationary position. The surgeon specified an injection volume of 50 µL and pressed the foot pedal to administer the exact amount of BSS to form a subretinal cavity. In both the manual and the remote groups, the surgeons were asked to inject approximately 1.5 disc diameters below the optic disc to maintain consistency between the two groups.

The endpoint of the injection was the observation of the formation of a round, grayish‐white elevated bulla; multiple unsuccessful attempts at injection to form a bulla; or a large amount of retinal hemorrhage interfering with the surgical field of view. At the end of the operation, tobramycin dexamethasone ophthalmic ointment was applied to the operative eye.

### Surgical Technique for Removing the Intraocular Foreign Body

To assess the instrumentation versatility of our remote robotic system, we integrated a pair of retinal forceps into the robotic arm for removing sutures (Alcon, 10–0 nylon suture) near the optic disc of porcine eyes. The sutures were cut into 2 mm long pieces and placed on the retinal surface of enucleated porcine eyes that had undergone vitrectomy. The ability was assessed to remove foreign bodies at two levels of complexity: easy (in which the sutures were situated in the flat regions of the retina) and difficult (in which the sutures were located within folds of the retina).

### Hybrid Parallel‐Serial Micromanipulator

The HPSM combined elements of both parallel and serial manipulator structures to provide greater flexibility and precision in controlling the position and orientation of objects or tools at a very small scale. In a parallel manipulator, paired micromotors driven the precise and stable translation or rotation of an end‐effector by controlling the relative motions of rods. Serial manipulators consisted of a chain of serially connected links and joints, where the motions were typically carried out sequentially from the base to the end effector. The HPSM had five DOFs, including three translational DOFs along the XYZ axes and two rotational DOFs. However, when it operated within the constraints of the RCM strategy, the available DOFs were limited. Specifically, there was only one translational DOF along the Z‐axis, and the two rotational DOFs were in the X‐Z and Y‐Z planes. This limitation arose because RCM constraints restricted translation within the tangent plane to prevent expansion of the scleral puncture port or the development of potential complications.

### Remote Center‐of Motion Strategy

A software‐based RCM strategy was employed to enable fine movements of the surgical instruments around a defined RCM point, thereby enhancing the precision and dexterity of the system within the relatively confined vitreous cavity. First, the position and posture of the surgical instrument are calculated according to the positive kinematics of the robot and the positions of the five motors. Afterward, equations of the inverse kinematics of the robot are derived from the positive kinematics, allowing us to determine the displacement of each motor on the basis of the changes in position and posture of the surgical instruments as directed by the controller at the surgeon site.

For the forward kinematics, we calculate the joint translation of motor 3 (q3) and motor 4 (q4) as *L_a_* and the joint translation of motor 1 (q1) and motor 2 (q2) as *L_b_*:

(1) $$L_{a}=\frac{q_{3}+q_{4}}{2}$$

(2) $$L_{b}=\frac{q_{1}+q_{2}}{2}$$

where *q_n_* denotes the displacement of the rod driven by motor *n*. In addition, the rotational angles of the tip of the surgical tool in the X‐Z and Y‐Z planes, denoted as θ_1_ and θ_2_, are generated by the different displacements of the rods driven by paired motors in a parallel manipulator:

(3) $$\theta_{1}=\arctan\left(\frac{q_{4}-q_{3}}{d_{m}}\right)$$

(4) $$\theta_{2}=\arctan\left(\frac{q_{2}-q_{1}}{d_{m}}\right)$$

where *d_m_* denotes the distance between the two hinge shafts of the parallel joint in the Z direction driven by paired motors in the parallel manipulator.

Additionally, the projected coordinates of the surgical instrument in the X, Y, and Z axes can be represented as:

(5) $$\begin{array}{ccc} & & x=L_{a}+h\;\cos\left(\theta_{1}\right)-\sin\left(\theta_{1}\right)\left(L_{\mathrm{tool}}\cos(\theta_{2})+h\;\sin(\theta_{2})\right)\\ & & -q_{5}\sin\left(\theta_{1}\right)\sin\left(\theta_{2}\right) \end{array}$$

(6) $$\begin{array}{ccc} & & z=q_{5}\cos\left(\theta_{1}\right)\cos\left(\theta_{2}\right)+\cos\left(\theta_{1}\right)(L_{\mathrm{tool}}\cos(\theta_{2})+h\;\sin(\theta_{2}))\\ & & +h\;\sin(\theta_{1}) \end{array}$$

(7) $$y=L_{b}-L_{\mathrm{tool}}\sin\left(\theta_{2}\right)+h\;\cos\left(\theta_{2}\right)-q_{5}\sin\left(\theta_{2}\right)$$

where h defines the length of the end effector and L_tool_ defines the distance from the end of the surgical tool to the center of its connection joint with the surgical robotic arm.

Combining Equations (3)–(7) and using them to determine the positions of the five motors separately, the Jacobian matrix can be calculated. Afterward, the inverse of the Jacobian matrix can be used to calculate the inverse kinematics as follows:

(8) $$q=J^{†}\cdot p$$

where *q* represents the locations of the five motors, J^†^ represents the generalized inverse of the Jacobian matrix, and the *p* represents the position and posture of the tool.

### Tremor‐Filtering Module

The tremor‐filtering module sampled the physiological tremor characteristics of the surgeon's hand using a simulated surgical system (Figure S4, Supporting Information). The simulator was designed to guide the operator to use the same master controller employed in actual procedures. Tremor data were collected under specific conditions: complete static posture, unidirectional movement (actuating a single encoder), and compound motion (actuating multiple encoders). Following data collection, the module compared the deviation between the guided trajectory and the actual trajectory for different motion types, and the 90^th^ percentile of the deviation values for each encoder was taken as the user‐specific filtering threshold. These customized thresholds were subsequently applied to suppress physiological tremors during surgical operations. In practical use, the system determined whether the current state corresponds to unidirectional movement or compound motion based on the new encoder position. Specifically, the variations of the two rotary encoders were normalized and compared: if the position fell within 2*n* × 45° ± 16.5° (*n* ∈ [0,3]), the state was classified as unidirectional movement; if within (2*n* + 1) × 45° ± 16.5° (*n* ∈ [0,3]), it was classified as compound motion; and if within the transition dead zone of (2*m* + 1) × 22.5° ± 6° (*m* ∈ [0,7]), the previous state was maintained. A dead zone of 12° was introduced between states to prevent frequent switching and instability near boundaries. Once the state was determined, the corresponding threshold set was applied. The system then evaluated whether the changes in the two encoder values exceeded their respective thresholds, and only when at least one encoder exceeded its threshold was the corresponding encoder value updated and transmitted.

### Network‐Adaptive Module

To mitigate the risks associated with network instability, a network‐adaptive module was implemented. This module continuously monitored network conditions and automatically adjusted the robot's trajectory when significant latency or data anomalies were detected, thereby preventing unexpected and potentially hazardous motions. A baseline latency value was first established by averaging delays over 20 remote connections, with a safety threshold set at twice this value to suspend operation in cases of excessive delay—reducing the risk of erroneous robot actions due to network fluctuations. During surgery, the system continuously tracked network performance. In case of disconnection, the patient‐side robot entered a safely paused state, permitting either reconnection or local takeover depending on the situation; operation resumed only after stable communication was restored. Moreover, if abnormal data transmission from the surgeon console was detected—characterized by significant deviations from predefined constraints—the system classified the movement as overspeed, immediately halted operation, and required explicit user confirmation before proceeding.

To ensure the safe and reliable execution of surgery, network tests were conducted under four scenarios: a) measurement of latency in robotic arm movement, injection, and needle withdrawal under normal network conditions; b) evaluation of command execution delay after reconnection following a transient network disconnection; c) evaluation of command execution delay after reconnection following a prolonged network disconnection; and d) assessment of whether the surgical robot exhibited high‐risk misoperations under moderate‐to‐high latency conditions. The results show that under normal network conditions, the average delay of the surgical robotic arm was 43.92 ms, the injection delay was 15.20 ms, and the needle withdrawal delay was 26.58 ms. Following both transient and prolonged network disconnections, the system successfully reestablished stable connections, with post‐reconnection delays remaining largely consistent with those observed under normal operation. Under artificially induced moderate‐to‐high latency conditions, when the surgeon performed standardized procedures for surgery, injection, and needle retraction, the actual operations executed by the robot were fully consistent with the standardized commands, with no occurrence of high‐risk misoperations.

In addition, to mitigate the impact of network fluctuations on the motion smoothness of the surgical robot, a dynamic command buffering mechanism was implemented on the patient‐side system. Incoming operational commands were first stored in a buffer and subsequently executed at the motor broadcast frequency of the robotic arm. The buffer management system continuously calculated the average delay and jitter based on the five most recent commands. The buffer depth was dynamically adjusted according to network conditions, set to the average delay plus twice the jitter value. Operation was automatically paused if the current delay exceeded 100% of the baseline level. Upon receipt of a stop or needle retraction command, such instructions were prioritized to the front of the buffer for immediate execution. After completing the prioritized command, the system cleared all remaining buffered commands, entered a paused state, and awaited new instructions from the surgeon.

### Dedicated Module for Remote Surgery

To adapt the original local surgical robot for telesurgery, dedicated modules were developed for remote operation. The module enabled real‐time visualization of the status of the remote robotic system and facilitated functional switching between different instruments, such as the opening/closing of micro forceps and the precise quantitative injection of microneedles, thereby ensuring the instrument adaptability and operational accuracy of the remote system for surgeons. In practical applications, surgeons configured parameters via the module interface according to surgical requirements, including manual/auto injection mode, total injection volume, single injection volume, and injection speed, which supported the successful completion of remote surgical procedures (Figure S5, Supporting Information). For remote signal communication, encoder data with a resolution of 1000 Hz were processed by aggregating every 10 data points into a single movement command. Outliers were removed during processing, and the remaining data, combined with a motion‐scaling factor, were used to determine tool velocity. Finally, the end‐effector velocity and injection motor control data were packaged and transmitted to the patient side.

### Stability, Accuracy, and Safety of the Robotic System

At the surgeon site, operational data underwent processing steps including tremor filtering and motion adjustment to enhance system stability. The program consolidated multiple adjacent encoder readings into single data points, thereby reducing the data acquisition rate to mitigate robot jitter induced by high‐frequency responses. Additionally, the deadband for encoder sampling was set at the boundary between the adjacent front and rear values because when the controller closed the operational boundary, hand trembling signals might be collected, so this strategy helped eliminate the signals from the tremor. Furthermore, to account for the requirements of speed scaling, the scaling ratios for the depth translation speed and rotation speed were set by the system to reduce the motion and angular range corresponding to manual manipulations of the surgical robotic arm proportionally, thereby improving the operational precision and stability. At the patient site, the HPSM had no singularity in its workspace, meaning that every position and posture assumed by the surgical tool could correspond to a single motor equation solution. This property ensured trajectory consistency and prevented sudden motor acceleration to maximum speed when the end of the tool approached the singularity. In addition, based on dynamic calculations, the robot incorporated counterweights in each parallel unit to self‐balance its own weight, which improved tool stability and reduced the risk of loss of the robot in use.

The safety monitoring mechanism was designed to cover three critical aspects: network connectivity, device status, and surgical operation. A three‐tier hierarchical protocol was implemented to address potential issues: Level 1 (Warning) involved fine‐tuning movements while logging event timestamps and corresponding data; Level 2 (Alert) required pausing control and notifying the surgeon of the current status; Level 3 (Emergency Stop) consisted of halting all robotic movements completely and enabling local patient‐side takeover to continue the procedure.

During network fluctuations or transient high latency below the predefined threshold, Level 1 handling was activated: the end‐effector continued to operate smoothly using buffered data, while network fluctuation metrics, command history, and robot motion data were recorded for subsequent analysis. If latency exceeded the set threshold, Level 2 handling was triggered: control was paused while network conditions were continuously monitored; remote operation could be resumed once stability was restored. In the event of disconnection, local backup networks were activated to attempt reconnection. If all network connection failed, the system entered Emergency Stop (Level 3), halted all movement, and switched to local control for needle retraction or surgery completion. Safety monitoring mechanism monitored robot internal data, including motor heartbeat signals and operational status, as well as the degree of RCM point drift. Upon status abnormality, the system paused and issued an alert (Level 2), awaiting clearance by the support engineer; severe cases triggered Emergency Stop (Level 3) with local takeover for instrument retraction or procedure continuation. If excessive movement speed or target positions beyond predefined boundaries were detected, the system initiated a pause (Level 2) and required explicit confirmation before resuming operation.

Ten in vivo robot‐assisted procedures were conducted in rabbits to validate the feasibility and stability of the robotic system in a local setting. The local robot‐assisted subretinal injection procedures were performed with the same surgical setup and robotic system as the subsequent remote procedures. The only difference was that the robot was operated directly on‐site by experienced ophthalmic surgeons using a standard surgical microscope. A comprehensive overview of these local procedures, including surgical duration, tremor amplitude, and postoperative outcomes, is summarized in Table S1 (Supporting Information), providing essential baseline validation for remote robotic experiments. Across all 10 cases, subretinal bleb formation was achieved on the first attempt without any lens or retinal complications. Retinal reattachment was observed in 7 out of 10 eyes (70%) by postoperative Day 1, and in all eyes (100%) by Day 3, indicating favorable surgical and healing outcomes.

### Remote Digital Stereoscopic Display System

The remote digital display system comprises several key components, including microsurgical image capture devices, video codec devices, dual image processing devices, and binocular stereo vision displays. Real‐time microsurgical images are captured with a dual‐camera setup (consisting of a 1/1.8 in. high‐sensitivity chip capable of capturing videos at 3840*2160@60P resolution) mounted on the objective lens of the surgical microscope (OPMI VISU 200, Carl Zeiss, Germany). The captured videos are converted to high‐definition multimedia interface (HDMI) signals while maintaining the same 3840*2160@60P resolution and then encoding seamless network transmission. The encoded signals are subsequently streamed through a 5G network slicing infrastructure (100 Mb/s, China Telecom Co., Ltd.) to the surgeon site, where the dual video signals are subsequently decoded as HDMI signals and reconstructed into a stereoscopic image via a computer with the following specifications: central processing unit (CPU)—Intel i5 8400, random access memory (RAM)—16GB DDR4, and graphics processing unit (GPU)—NVIDIA GeForce GTX1050Ti. Binocular stereo‐images are displayed by a digital visualization system tailored to microsurgery (SDP3D5500, a 55‐inch display with a resolution of 3840*2160@60P). The display is positioned at a distance of approximately 2 m in front of the microsurgeons, as recommended by previous studies, to ensure the best visual experience.^[^ ^33^, ^34^ ^]^ To perceive the stereo vision presented on the display, the surgeons are required to wear film pattern slowing glasses. This system allows surgeons at the surgeon site to perceive the details of the surgery and the depth information within high‐definition and low‐latency stereoscopic images. This innovative system empowered surgeons to have a detailed view of the surgery and make precise adjustments despite being physically distant from the operating room.

### Data Collection and Outcome Evaluation

A successful subretinal injection was defined as the formation of a grayish‐white, rounded, elevated vesicle without visible retinal tears or severe retinal hemorrhages. Intraoperative complications were recorded via surgical video recording, and the retinal conditions of the rabbit eyes were assessed with OCT imaging (Spectralis HRA, Heidelberg) and fundus photography (TR300, TOMEY) conducted 3 h and 1, 3, 7, and 14 d postoperatively to record retinal recovery and postoperative complications.

The primary outcome of this study was the first‐attempt success rate of subretinal injection, that is, the frequency at which the retina was punctured only once to form an injection hole during the same procedure, and subretinal injection was successfully completed. Other outcomes included the total operative time, injection time, incidence of intraoperative complications, incidence of postoperative complications, and duration of retina and injection hole recovery. Two retinal specialists who were not involved in the surgery or postoperative examinations independently and blindly evaluated complications and recovery time by assessing the surgical videos and images. When a consensus could not be reached, a third, more senior retinal specialist was introduced to arbitrate the results. For foreign body retrieval, the success rate and duration of the procedure were evaluated by examining the surgical videos. Since foreign body retrieval was carried out on enucleated porcine eyes, no intraoperative or postoperative complications were recorded.

### Statistics Analysis

The means and 95% CIs were used to describe normally distributed data, and the unpaired t test was used to assess differences in these data between the remote and manual groups. Outcomes that did not conform to a normal distribution were expressed as the median and IQR and were compared between the groups with the Mann‒Whitney U test. The sample size for each interventional arm required to achieve 80% statistical power with a 2‐sided test at an *α* = 0.05 with the Z test for the first‐attempt success rate was at least 22 eyes. Differences in the success rates between the remote robotic and manual surgery groups were assessed with the Z test. Statistical analyses were performed with SPSS Statistics V22.0 (IBM Corp., Armonk, NY), and a two‐sided *p* value less than 0.05 was considered statistically significant. Figures were plotted with GraphPad Prism (version 9.3.1) and Adobe Illustrator (version 2022).

## Conflict of Interest

The authors declare no conflict of interest.

## Author Contributions

A.X., Z.L., W.C., and Y.L. contributed equally to this work. Conceptualization: K.H. and H.L. Methodology: A.X., Z.L., W.C., Y.L., Z.L., L.J., H.S., W.L., M.A.N., K.H., and H.L. Investigation: J.L., Z.L., B.L., W.M., T.L., P.Y., X.W., D.L., X.C., X.Z., and H.L. Software: Y.L., H.L., R.S., and X.Z. Visualization: Y.H., M.L., and K.G. Data curation: A.X., Z.L., J.L., Z.Z., X.W., B.L., W.M., R.W., J.W. Funding acquisition: H.L., K.H., R.L., and W.C. Supervision: A.X., K.H., and H.L. Writing: A.X., Z.L., W.C., Y.L., K.H., and H.L.

## Supporting information



Supporting Information

Supplemental Video 1

Supplemental Video 2

Supplemental Video 3

Supplemental Video 4

Supplemental Video 5

Supplemental Video 6

## Data Availability

The data that support the findings of this study are available from the corresponding author upon reasonable request.
