# Supplementary material for: Development of Teleoperated Robotic System for Remote Intraocular Microsurgery
Source: Adv Sci (Weinh). 2025 Nov 10;13(6):e09849. doi: 10.1002/advs.202509849 (PMC12866789; doi:10.1002/advs.202509849)
Supplement: Supplementary file 1 — Supporting Information [file ADVS-13-e09849-s007.docx]

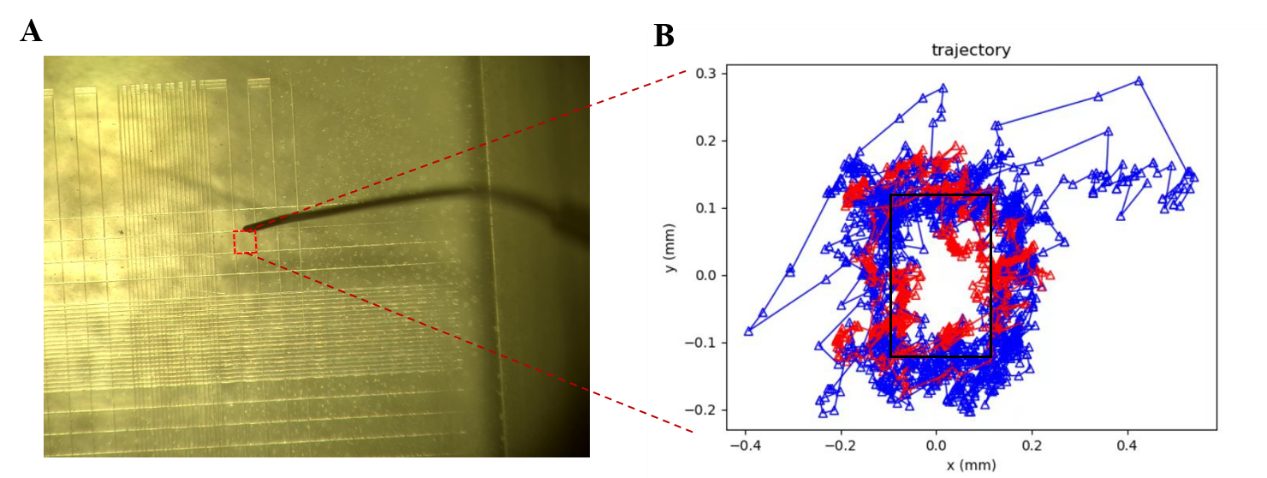


**Supplementary Fig. 1.** **Trajectories from a microsquare-tracing experiment.** (A) and (B) The red triangles indicate the needle tip’s trajectories when maneuvered by the remote robotic system, while the blue triangles represent trajectories under manual control. The black line represents the desired trajectory (a 0.25 mm×0.25 mm square).


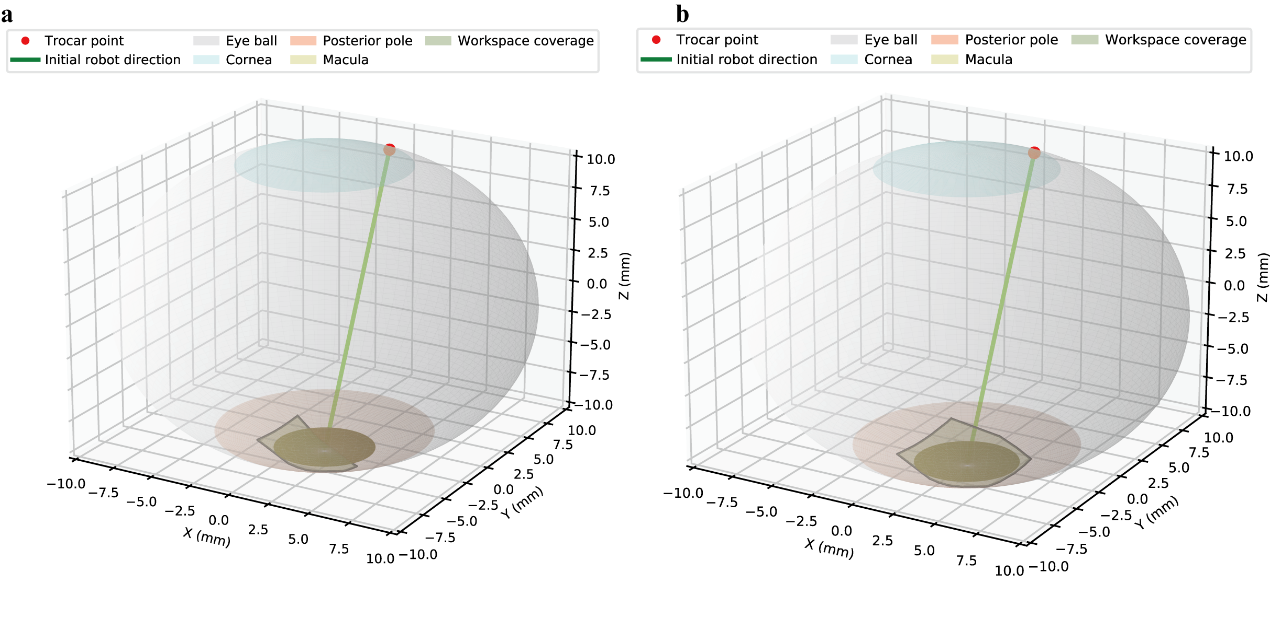


**Supplementary Fig. 2. Comparison of the coverage workspace in the macular region of the adult eyeball with an axial length of 24mm.** Estimated overlap ratio (overlap / macula) of (a) the robot without the assistance of additional motor (57.33 %, based on Monte Carlo sampling, 20000 samples) and (b) our remote robot system (100.00 %, based on Monte Carlo sampling, 20000 samples).


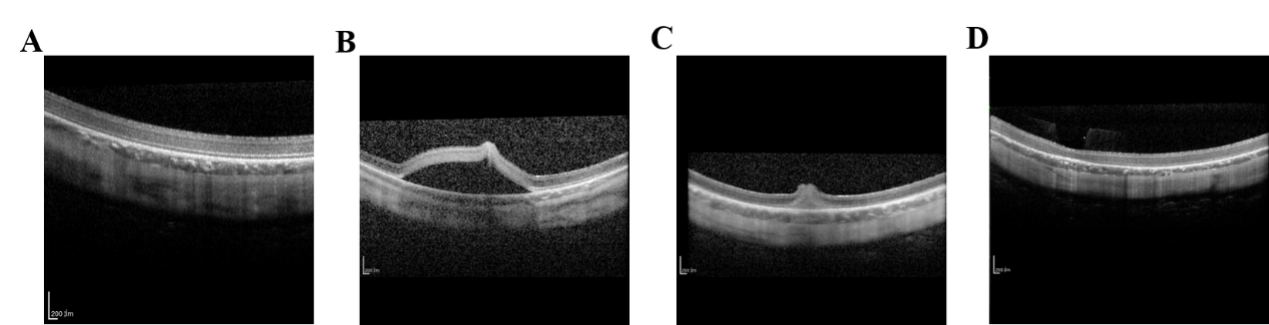


**Supplementary Fig. 3. OCT images of a pigmented rabbit eye before and after remote subretinal injection from Haikou to Guangzhou.** (A) The retina is flat and intact before surgery. (B) Three hours after surgery, balanced salt solution (BSS) is observed between the retina and the retinal pigment epithelium (RPE). (C) One day after surgery, the BSS is absorbed, and the injection hole is closed. (D) Seven days after surgery, the retinal structure has fully recovered.


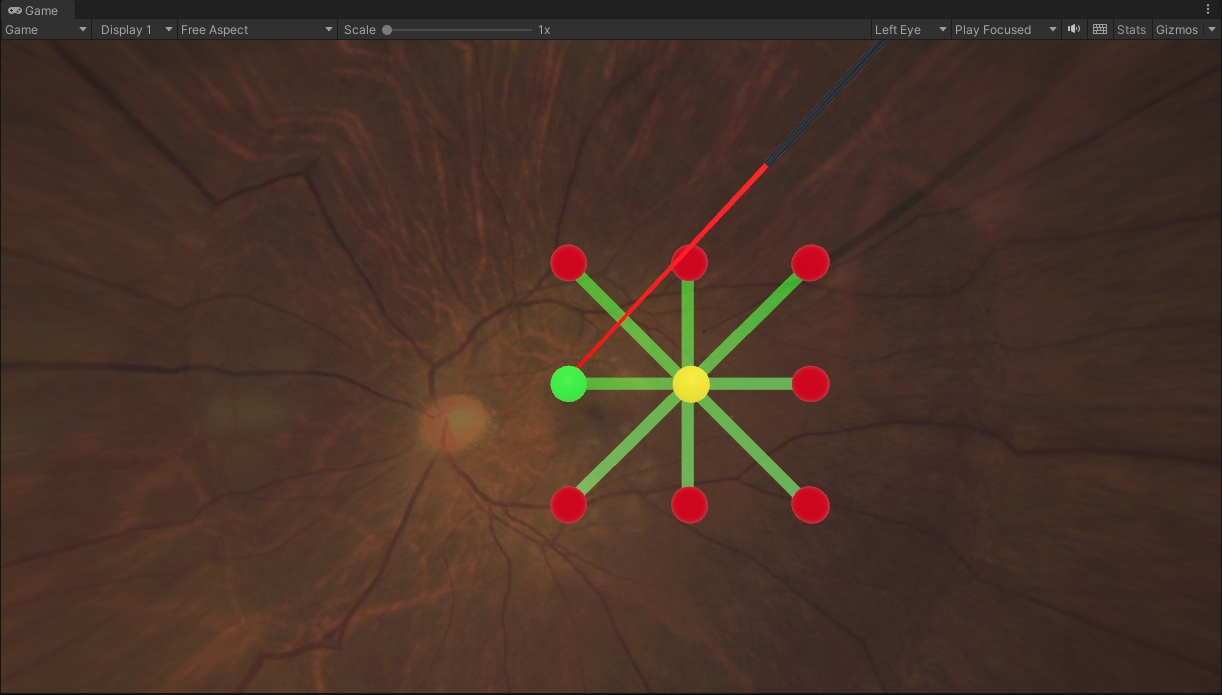


**Supplementary Fig. 4.** **Simulated physiological tremor acquisition and guidance system.** The simulation system collects hand tremor data from surgeons. The surgeons manipulate the end of the red line to follow the red dot and the green guide line to perform three types of movements, including complete stillness, unidirectional movement, and compound movement. During the operation, the handle held by the surgeon synchronously collects the data of the surgeon's hand tremors. After the acquisition is completed, customized thresholds for different movements are set for surgeons based on the collected physiological tremors, which are used to filter out physiological tremors during actual surgeries.


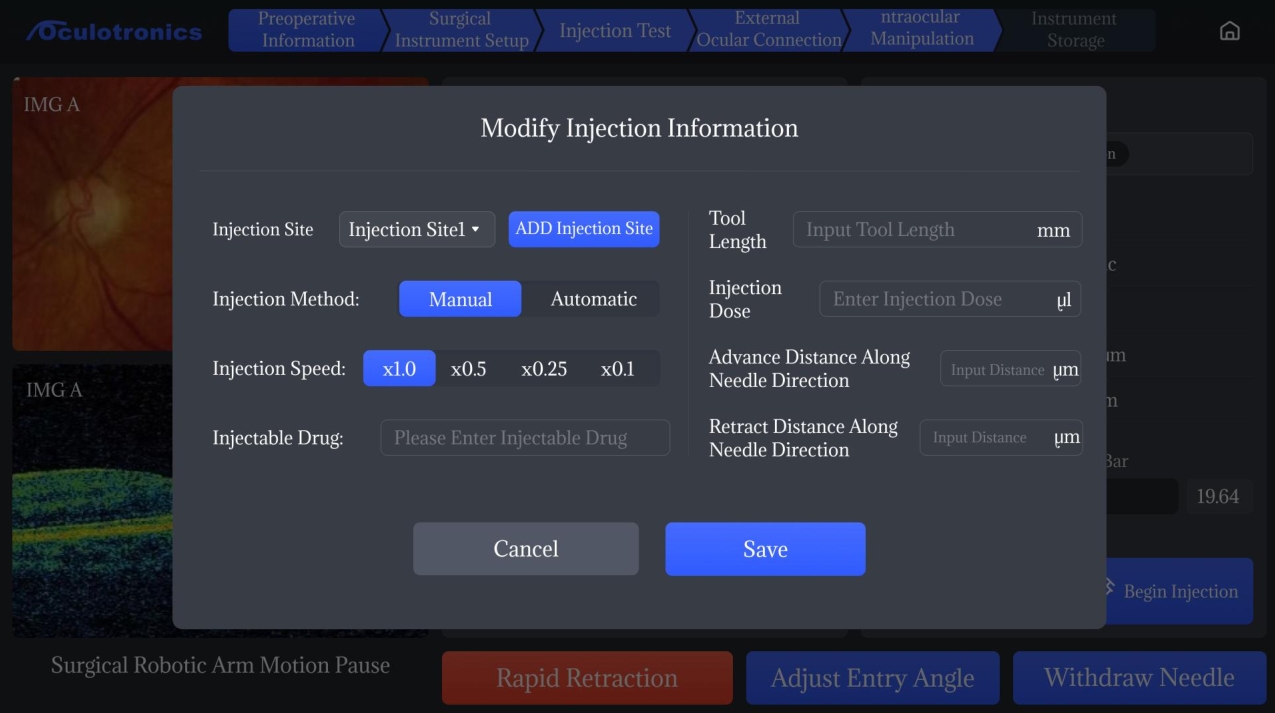


**Supplementary Fig 5. Injection system interface for remote operation.**

**Supplementary Table 1: Summary of Robot-Assisted Subretinal Injection – Local Preclinical Validation**

| Outcome (N=10) | Rate or Mean | SD | Median | IQR |
| --- | --- | --- | --- | --- |
| First-attempt Success rate (%, N) | 100% (10/10) | - | - | - |
| Complication rate (%, N) | 0% (0/10) | - | - | - |
| Retinal reattachment on Day 1 (%, N) | 70% (7/10) | - | - | - |
| Retinal reattachment on Day 3 (%, N) | 100% (10/10) |  |  |  |
| Total surgery duration (s) | 708 | 185 | 660 | 210 |
| Injection duration (s) | 207 | 52 | 180 | 60 |
| Maximum tremor amplitude (μm) | 122.7 | 43.6 | 118.2 | 44.6 |
| Mean tremor amplitude (μm) | 36.8 | 21.0 | 28.0 | 26.9 |
